# Aortic displacement and hemodynamics are abnormal in patients with Marfan syndrome: A combined four-dimensional balanced steady-state free precession and four-dimensional flow cardiac magnetic resonance study

**DOI:** 10.1016/j.jocmr.2026.102731

**Published:** 2026-04-20

**Authors:** Daan Bosshardt, Renske Merton, Aart J. Nederveen, Roland R.J. van Kimmenade, Moniek G.P.J. Cox, Arthur J.H.A. Scholte, Eric M. Schrauben, Daniëlle Robbers-Visser, Maarten Groenink, Pim van Ooij

**Affiliations:** a Radiology and Nuclear Medicine, Amsterdam University Medical Center, Amsterdam, the Netherlands; b Amsterdam Cardiovascular Sciences, Amsterdam, the Netherlands; c Cardiology, Amsterdam University Medical Center, Amsterdam, the Netherlands; d Zuyderland Medical Center, Heerlen, Sittard-Geleen, the Netherlands; e Cardiology, University Medical Center Groningen, Groningen, the Netherlands; f Cardiology, Leiden University Medical Center, Leiden, the Netherlands

**Keywords:** 4D bSSFP, 4D flow CMR, Marfan syndrome, Arterial stiffness

## Abstract

**Background:**

Marfan syndrome (MFS) patients with a history of aortic root surgery (ARS) are at increased risk of type B aortic dissection, possibly because the noncompliant graft fails to absorb systolic forces, leading to undampened flow in the proximal descending aorta (pDAo). In this study, we investigated the magnitude and location of abnormal aortic displacement and wall shear stress (WSS) using cardiac magnetic resonance (CMR) imaging.

**Methods:**

We examined 82 MFS patients (32 with ARS, 34 **±** 8 years, 36 women) and 45 age- and sex-matched controls, all undergoing four-dimensional high-resolution balanced steady-state free precession and flow CMR. Peak displacement and WSS were calculated using automated aortic segmentations. Heatmaps were created to identify and quantify regions with abnormal displacement and WSS. The surface areas of abnormal displacement and WSS were quantified in four aortic regions. The Wilcoxon signed-rank test was used for comparison of abnormal CMR parameter quantification, and Pearson correlation was used to assess correlations between displacement and WSS.

**Results:**

WSS was higher in the proximal (p < 0.001) and distal ascending aorta (Aao) (p = 0.031) of ARS MFS patients compared to native MFS patients. ARS MFS patients had significantly larger surface areas of decreased displacement (p < 0.001; present in 97% (31/32) of ARS MFS patients) and increased WSS (p < 0.001; present in 100% (32/32) of patients) in the proximal Aao. There was a trend toward a larger surface area of increased displacement in the pDAo of ARS MFS patients (p = 0.062; present in 72% (23/32) of patients), mainly located in the outer pDAo. pDAo displacement was positively correlated with pDAo WSS (Pearson r = 0.46 [95% confidence interval: 0.13, 0.70], p = 0.008).

**Conclusion:**

Aortic displacement and flow characteristics are abnormal in MFS patients, both with and without a history of ARS. ARS MFS patients exhibit distinct features in both the AAo and descending aorta (DAo), particularly in the proximal DAo, which is the region susceptible to type B aortic dissections.

## Graphical abstract

**Figure:**
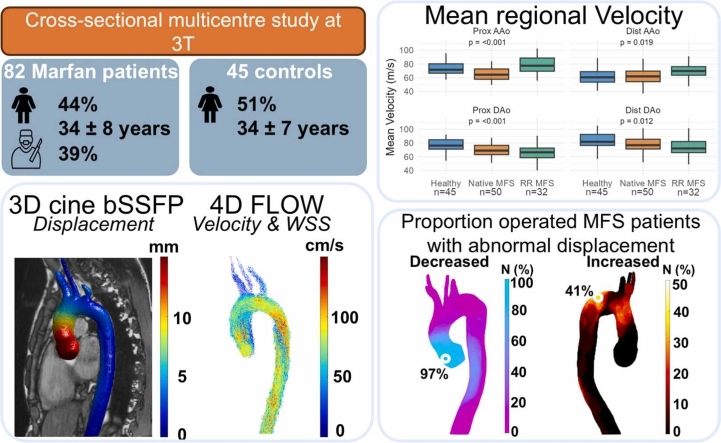

## 1 Introduction

Patients with Marfan syndrome (MFS) are at risk of developing aortic aneurysms and dissection due to an autosomal dominant mutation in the *FBN1* gene affecting all connective tissue [1]. While the introduction of aortic surgery techniques to replace the aortic root has decreased the incidence of aortic dissections affecting the ascending aorta (AAo) (type A aortic dissections (AAD)), the unprotected descending aorta (DAo) is still at risk for type B aortic dissection (TBAD). Where aortic diameter is a strong predictor for AAD, 88% of the TBADs occur in normal or mildly dilated aortas [2], [3], [4]. Currently, no markers exist that can predict TBAD in MFS; thus, a better understanding of the pathophysiological process underlying TBAD development is warranted.

MFS patients with a history of aortic root surgery (ARS) are at increased risk of TBAD [3], [5]. It has been hypothesized that changes in aortic hemodynamics after surgery might play a role in TBAD development in these patients, as the noncompliant graft might be unable to absorb systolic forces, leading to undampened flow in the DAo [6].

Three-dimensional (3D) phase-contrast cardiovascular magnetic resonance imaging (four-dimensional [4D] flow CMR) is a suitable tool to assess aortic hemodynamics in health and disease [7]. The technique not only allows for the evaluation of aortic blood flow and velocities but also for the quantification of parameters such as wall shear stress (WSS) and pulse wave velocity (PWV). Abnormal WSS has previously been associated with risk factors for aortic complications in bicuspid aortic valve and MFS [8], [9], [10], [11]. PWV is an indirect measurement of arterial stiffness, which is associated with adverse cardiovascular events in the general population and is increased in patients with MFS [12], [13], [14].

We have recently proposed 3D aortic displacement during the cardiac cycle as a potential new measurement for arterial stiffness derived from a 3D cine balanced steady-state free precession (4D bSSFP) CMR sequence [15], [16]. We have shown that 3D displacement is reduced in the AAo of patients with MFS compared with healthy volunteers (HVs). Combining data on flow and displacement in one examination allows for the comprehensive evaluation of the aortic hemodynamics and biomechanics and might provide valuable information on the pathogenesis of aortic dilatation and dissection.

Therefore, the aim of the current study was to 1) compare regional 4D bSSFP derived displacement and 4D flow CMR parameters of patients with a history of ARS (ARS MFS) to patients without previous root surgery (native) and with HVs; 2) to identify regions where displacement and hemodynamics are abnormally increased or decreased and directed, and 3) to assess the relationship between 4D bSSFP and 4D flow CMR parameters.

## 2 Methods

### 2.1 Patient characteristics

The local ethics boards approved this study, and written informed consent was obtained from all participants. Patients and HVs were enrolled from February 2023 to June 2024. Patients were identified in four Dutch hospitals with a specialized multidisciplinary Marfan screening clinic. Eligible patients were aged 18–50 years, diagnosed with MFS according to the revised Ghent criteria, with a known pathological *FBN1* variant, without a contraindication for CMR, without aortic surgery beyond the AAo, and without an internal cardiac defibrillator or pacemaker, as previously described [1], [14]. Age- and sex-matched HVs without a history of cardiovascular disease were recruited via social media and a local recruitment platform. Patients over 50 years old were not eligible to limit the influence of age-related cardiovascular disease on the observations. All study examinations were performed at one center (Amsterdam UMC). Three groups were defined: HVs, native, and ARS MFS patients.

### 2.2 CMR acquisition and reconstruction

All participants underwent imaging of the thoracic aorta on a 3T scanner (Philips Healthcare, Best, the Netherlands) equipped with a 16-channel anterior coil and 12-channel posterior coil.

A 4D bSSFP scan was acquired, immediately followed by 4D flow CMR, both performed in free-breathing and without contrast agent administration, using the same prescribed field of view (FOV) [15], [17]. The orientation and FOV size were tailored to the patient's size to cover the anterior-posterior chest wall dimension and right-left extent of the thoracic aorta. Both scans were acquired using an in-house developed pseudo-spiral Cartesian acquisition scheme and reconstructed using a compressed sensing pipeline using the Berkeley Advanced Reconstruction Toolbox (BART, Berkeley, California), Matlab R2022b (The Mathworks Inc., Natick, Massachusetts), and ReconFrame 4.2.0 (Gyrotools, Zurich, Switzerland) [18], [19].

Scan parameters of the 4D bSSFP scan were FOV = 256 × (256 − 320) × [70 − 88] mm^3^, with the latter two varying dependent on patient’s size, slice oversampling factor = 1.70–2.14; acquired/reconstructed spatial resolution = (1.6/1.0 mm)^3^ isotropic, reconstructed to 30 cardiac phases, repetition time (TR)/echo time (TE)/ flip angle (FA) = 2.90 ms/1.44 ms/40°, with a sinc-Gauss radiofrequency pulse shape with one sinc-period [20]. The median (interquartile range [IQR]) scan time was 4:21 min (4:15–4:22), depending on the FOV and arrhythmia rejection, with a mean acceleration factor of 19.0 **±** 2.7. Electrocardiography or photoplethysmography was used for retrospective cardiac binning. To correct for respiratory motion, automated self-gating was used to retrieve the respiratory signal and retrospectively register and average four respiratory bins to end-expiration [15].

The 4D flow CMR scan parameters were TR/TE/FA = 4.7ms/2.5ms/4° with velocity encoding = 150–250 cm/s, slice oversampling factor = 1, with the same spatiotemporal resolution as the 4D bSSFP scan. The median scan time was 7:51 min (IQR: 6:48– 9:21), depending on FOV size and arrhythmia rejection. Respiratory state information was acquired using a lung-liver navigator and used for retrospective respiratory gating as previously described, resulting in a mean (standard deviation [SD]) acceleration factor of R 13.8 **±** 1.4 [17]. Electrocardiography or photoplethysmography was used for retrospective cardiac gating. Postprocessing involved 4D automatic Laplacian phase unwrapping and correction for background phase offsets [21], [22]. Time-averaged phase contrast magnetic resonance angiogram (PCMRA) images (phase contrast magnitude images multiplied by absolute velocity) were created.

A clinically used mid-diastolic 3D mDixon scan with isotropic spatial resolution of 0.75 mm was acquired to measure the aortic diameter in cross-sectional planes manually placed on four predefined levels: the aortic root (at the level of the sinus of Valsalva, maximum of cusp-to-cusp distance); the AAo and proximal descending aorta (pDAo) at the level of the pulmonary artery bifurcation, and at the level of the diaphragm.

### 2.3 CMR postprocessing

#### 2.3.1 Segmentation

Time-resolved aortic segmentations of the 4D bSSFP scans were created with an nnU-net using previously published settings [16], [23]. The network was trained on 87 manual aorta segmentations of varying cardiac phases of 74 randomly selected participants enrolled in the study (13 volunteers and 61 MFS patients).

Time-averaged aortic segmentations for the 4D flow MRI-derived PCMRA were created with an nnU-net trained on manual segmentations from randomly selected scans of seven MFS patients and four volunteers enrolled in the current study and 106 scans of 60 MFS patients and 25 volunteers enrolled in a previous study [24]. All 4D bSSFP and 4D flow CMR segmentations were manually evaluated by D.B. and manually corrected, if necessary (n = 1 for the 4D bSSFP scan, n = 33 for the PCMRA segmentations).

#### 2.3.2 4D bSSFP image processing

The time-resolved 4D bSSFP segmentations were used to create 3D displacement maps by non-rigid registration of the cardiac phase at end-diastole to the segmentations of other cardiac phases, followed by calculating the Euclidean distance per surface point of the segmentation, as previously described [16], [25]. The timeframe with the maximum average displacement was used for analysis.

#### 2.3.3 4D flow CMR image processing

WSS vectors were calculated at the aortic wall by deriving 3D spatial velocity gradients perpendicular to the vessel wall, using three points along the inward normal of the vessel wall, spanning 50% of the lumen diameter [26]. 3D WSS was calculated at peak systole, defined as the timeframe in which the spatially averaged velocity magnitude within the segmented aortic volume was highest.

#### 2.3.4 Parameter quantification

The aorta was subdivided into four manually placed regions of interest (ROIs) in the thoracic aorta on the PCMRA segmentations: the proximal ascending aorta (pAAo), from the aortic annulus to the level of the pulmonary artery bifurcation; the distal ascending aorta (dAAo), from the pulmonary artery bifurcation to the brachiocephalic trunk; the pDAo, from the left subclavian artery to the level of the pulmonary artery bifurcation; and the distal descending aorta (dDAo), from the pulmonary artery bifurcation to the diaphragm.

Next, the 4D flow CMR ROIs were projected onto the 4D bSSFP scan and adjusted to the correct position if any misalignment was detected upon inspection (n = 11).

4D flow CMR-derived velocity and WSS values, and 4D bSSFP-derived displacement were averaged per ROI and used for analysis.

For PWV analysis, all data were reconstructed to a fixed 20 ms temporal resolution [27]. Subsequently, PWV was calculated using a previously described in-house developed Matlab application (Matlab version R2022b, The Mathworks Inc.) [28], [29], [30].

#### 2.3.5 Heatmaps and incidence maps

**Fig. 1:**
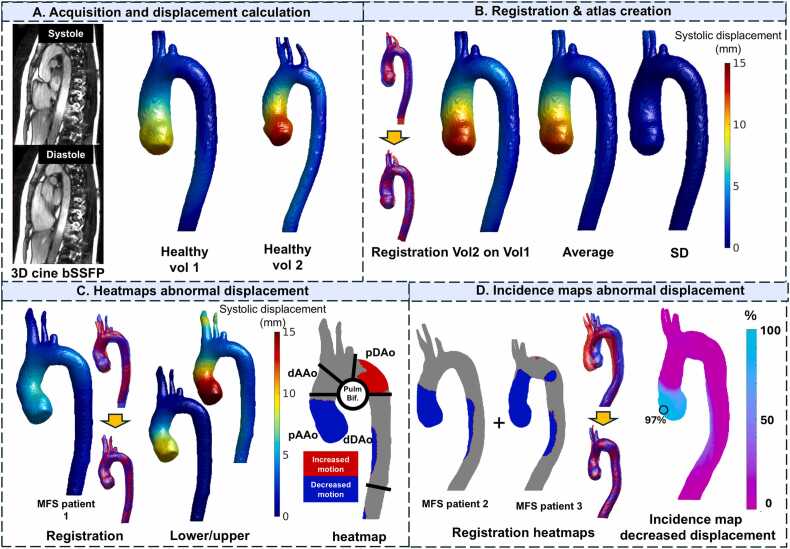
Creation of abnormal displacement heat and incidence maps. (A) Creation of peak-systolic displacement maps from the 3D cine balanced steady state free precession (bSSFP) scan for all volunteers; (B) registration and interpolation of displacement of volunteer 2 projected to the control geometry of volunteer 1, with the average displacement and standard deviation after interpolation of all 44 volunteers on the control geometry to create a displacement atlas; (C) registration of the control geometry to individual Marfan syndrome (MFS) patient geometry and interpolation of the displacement atlas, with subsequent creation of heatmaps identifying regions with displacement outside of the 95% confidence interval of the control atlas. (D) Registration of heatmap geometries to a control MFS geometry, interpolation of the individual MFS data to the control MFS geometry, and calculation of the proportion of patients with abnormal displacement at each location in the control MFS geometry. *3D* three-dimensional, *SD* standard deviation, *dAAo* distal ascending aorta, *pAAo* proximal ascending aorta, *dDAo* distal descending aorta, *pDAo* proximal descending aorta

Heatmaps localizing and quantifying regions with abnormal displacement were created as previously described for abnormal hemodynamics evaluation [24], [31]. The processing pipeline for the displacement analysis is presented in Fig. 1. First, 3D averages of normal displacement were created by registering the geometry of all included HVs to the geometry of the HV with the smallest mean distance to the geometries of other HVs (the control geometry template). Next, the peak displacement data of all HVs were registered using non-rigid registration and interpolated to this control geometry template [25]. After interpolation, the mean and SD map of all HVs were calculated, and 3D maps describing the 95% confidence interval (CI) of normal values of the aorta were created by adding and subtracting 1.96 times the SD map to the mean. By co-registering the control geometry template to the individual patient geometry and interpolating the created 95% CI maps, regions with abnormally increased or decreased displacement could be identified and quantified [32].

The 4D flow CMR heatmaps were created similarly, and as previously described [24]. Furthermore, WSS vector heatmaps were created to identify abnormal WSS vector direction, defined as vector angle differences higher than 120° compared to the average volunteer vector direction [33].

Subsequently, incidence maps were created by registering all heatmaps to a patient geometry template derived from the 4D bSSFP scan segmentation with the smallest average distance to the segmentations of the other patients and expressing the proportion of patients with abnormal displacement or hemodynamics at each wall point [31].

Differences in pre- and post-registration displacement and WSS were calculated over all ROIs combined.

### 2.4 Other parameters

Data on baseline characteristics and medication use were retrieved from the electronic patient records and a questionnaire.

Peripheral blood pressure and heart rate were recorded non-invasively from the brachial artery using a Microlife BP A100 Plus (Microlife AG, Widnau, Switzerland) oscillometric sphygmomanometer. The mean of two measurements before and one after the CMR examination was used for analysis.

### 2.5 Statistical analysis

Statistical analyses were performed using R version 4.4.3 (R Foundation for Statistical Computing, Vienna, Austria). A one-way analysis of variance (ANOVA) was used to compare differences in continuous baseline and CMR parameters between the three groups. As four ROIs were evaluated, ANOVA values <0.0125 for the CMR parameters were considered statistically significant, as defined by Bonferroni correction for multiple testing. For significant CMR parameters, pairwise comparisons were done using Tukey- Honestly Significant Difference tests. Categorical values were compared using a χ^2^ test. In each ROI and study group, Pearson correlations were performed to assess the correlation between displacement and each flow parameter (velocity, WSS, within the same ROI and global PWV). Univariable logistic regression was performed to assess determinants of abnormally increased pDAo displacement. A p-value of 0.05 was considered significant for baseline characteristics and pre-post registration comparisons, post-hoc Tukey Honestly Significant Difference tests, and, due to their exploratory nature, the correlation and regression analyses.

## 3 Results

### 3.1 Patient characteristics

**Fig. 2:**
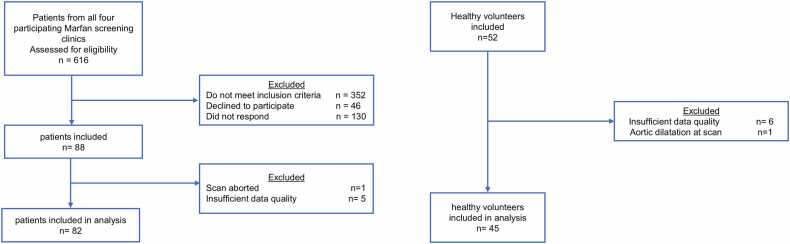
Inclusion flowchart

The inclusion flowchart is presented in Fig. 2. In total, 88 patients and 52 volunteers underwent the CMR. Data quality was insufficient for analysis in five patients and in six volunteers (six 4D bSSFP scans and five 4D flow CMR scans). In one MFS patient, the examination was terminated due to claustrophobia, and one volunteer was excluded due to previously undiagnosed aortic dilatation at the inclusion scan. Baseline characteristics of the 127 included participants are presented in Table 1.

**Table 1:** Baseline characteristics.

	Healthy volunteers (n = 45)	Native MFS patients (n = 50)	ARS MFS patients (n = 32)	p-value Group differences
Age at inclusion (years)	34±7	33±9	35±7	0.563
Sex (female)	23 (51%)	25 (50%)	11 (34%)	0.283
Height (cm)	178±12	188±10	193±9	**<0.001**
Weight (kg)	75±12	80±14	86±17	**0.006**
BSA (m^2^)	1.9±0.2	2.0±0.2	2.1±0.2	**<0.001**
Systolic BP (mmHg)	129±13	128±11	127±10	0.851
Diastolic BP (mmHg)	81±8	81±8	79±7	0.481
Mean arterial pressure (mmHg)	97±9	96±8	95±8	0.632
Pulse pressure (mmHg)	48±8	47±7	48±6	0.788
Heart rate (beats/min)	64±9	71±13	67±9	**0.017**
Betablocker use		18 (36%)	19 (59%)	0.065
ARB use		28 (56%)	18 (56%)	1.000
Betablocker and ARB		11 (22%)	10 (31%)	**0.047**
FBN1 mutation effect				0.102
Haploinsufficient		11 (22%)	14 (44%)	
Dominant negative		28 (56%)	14 (44%)	
Effect unknown		11 (22%)	4 (12%)	
Bicuspid aortic valve	0 (0%)	3 (6%)	0 (0%)	-
Root surgery procedure				
Valve-sparing			17 (53%)	
Bentall			12(38%)	
PEARS			3 (9%)	
Aortic diameters				
Root (mm)	34±4	43±5	-	**<0.001**
Ascending (mm)	31±4	31±4	32±5	0.610
Proximal descending (mm)	23±3	26±4	29±4	**<0.001**
Diaphragm (mm)	20±2	21±2	24±4	**<0.001**

*ARB* angiotensin II receptor blocker, *BP* blood pressure, *BSA* body surface area, *Native MFS* Marfan syndrome patient without a history of aortic root surgery, *PEARS* personalized external aortic root support, *ARS MFS* Marfan syndrome patient with a history of aortic root surgery

Variables presented as (mean ± SD) or n (%). p-values represent ANOVA, or t-test for continuous variables, and Fisher’s exact test for categorical variables. A p-value <0.05 was considered statistically significant

### 3.2 CMR parameters

Typical examples of displacement and velocity vector maps of an HV and a native, and ARS MFS patient are presented in Video 1. All peak displacement, velocity, and WSS values per ROI are presented in Table 2.

**Video 1:**
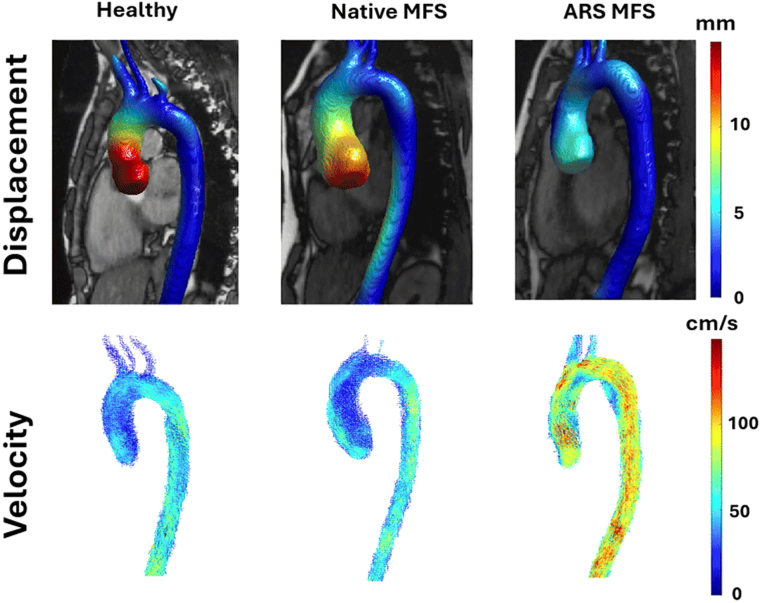
Example of displacement and flow in a healthy volunteer and two Marfan syndrome (MFS) patients without (native) and with (ARS) a history of aortic root surgery.

**Table 2:** Regional 4D bSSFP and 4D flow CMR parameters.

	Healthy (n = 45)	Native MFS (n = 50)	ARS MFS (n = 32)		Native MFS-healthy	ARS MFS-healthy	ARS MFS-native MFS
	Mean±SD	Mean±SD	Mean±SD	ANOVA p-value	Tukey p-value	Tukey p-value	Tukey p-value
*Displacement*							
Proximal ascending aorta (mm)	9.8±1.2	9.0±1.9	5.8±1.5	**<0.001**	0.052	**<0.001**	**<0.001**
Distal ascending aorta (mm)	4.5±1.0	4.6±1.2	4.3±1.2	0.412	-	-	-
Proximal descending aorta (mm)	1.5±0.4	1.4±0.4	1.6±0.5	0.156	-	-	-
Distal descending aorta (mm)	1.8±0.4	1.7±0.5	1.3±0.4	**<0.001**	0.620	**<0.001**	**0.001**
*Velocity*							
Proximal ascending aorta (cm/s)	73.2±9.5	65.1±9.4	78.4±12.1	**<0.001**	**<0.001**	0.075	**<0.001**
Distal ascending aorta (cm/s)	62.0±12.2	62.7±11.2	69.0±10.7	0.019	-	-	-
Proximal descending aorta (cm/s)	76.9±9.2	69.6±12.9	66.5±13.5	**<0.001**	**0.010**	**<0.001**	0.464
Distal descending aorta (cm/s)	82.6±11.9	76.3±13.3	74.2±14.3	**0.012**	0.053	**0.018**	0.766
*WSS*							
Proximal ascending aorta (Pa)	0.76±0.13	0.63±0.12	0.81±0.16	**<0.001**	**<0.001**	0.215	**<0.001**
Distal ascending aorta (Pa)	0.55±0.14	0.58±0.13	0.66±0.15	**0.005**	0.688	**0.004**	**0.031**
Proximal descending aorta (Pa)	1.07±0.18	0.88±0.23	0.76±0.20	**<0.001**	**<0.001**	**<0.001**	**0.035**
Distal descending aorta (Pa)	1.08±0.21	1.00±0.21	0.91±0.20	**0.003**	0.162	**0.002**	0.136
Pulse wave velocity (m/s)	6.6±1.4	9.0±3.5	10.0±2.7	**<0.001**	**<0.001**	**<0.001**	0.220

*4D* four-dimensional, *bSSFP* balanced steady-state free precession, *CMR* cardiovascular magnetic resonance, *Native MFS* Marfan syndrome patient without a history of aortic root surgery, *ARS MFS* Marfan syndrome patient with a history of aortic root surgery, *ANOVA* analysis of variance, *SD* standard deviation, *WSS* wall shear stress

Parameters expressed as mean ± SD. ANOVA p-values <0.0125 and Tukey post hoc p-values <0.05 were considered statistically significant

Displacement was significantly lower for ARS MFS vs HVs (5.8 ± 1.5 vs 9.8 ± 1.2 mm, p < 0.001) and vs native MFS patients in the pAAo (5.8 ± 1.5 vs 9.0 ± 1.9 mm, p < 0.001). In the dDAo, displacement was significantly lower for ARS MFS patients compared to both native MFS (1.3 ± 0.4 vs 1.7 ± 0.5 mm, p = 0.001) and HVs (1.3 ± 0.4 vs 1.8 ± 0.4 mm, p < 0.001), but there was no significant difference between native MFS and HVs (p = 0.620). Displacement was not significantly different between the three groups in the dAAo (p = 0.412) and the pDAo (p = 0.156).

Velocity in the pAAo was lower for native MFS patients vs HVs (65.1 ± 9.4 vs 73.2 ± 9.5 cm/s, p < 0.001) and vs the ARS MFS patients (65.1 ± 9.4 vs 78.4 ± 12.1 cm/s, p < 0.001). In the pDAo, velocity was lower for native MFS vs HVs (69.6 ± 12.9 vs 76.9 ± 9.2 cm/s, p = 0.010) and for ARS MFS patients compared to HVs (66.5 ± 13.5 vs 76.9 ± 9.2 cm/s, p < 0.001), but no difference was observed between the two MFS categories (p = 0.464).

In the dDAo, velocity was significantly higher for HVs vs ARS MFS patients (82.6 ± 11.9 vs 74.2 ± 14.3 cm/s, p = 0.018), but not between MFS categories (p = 0.766) or between native MFS patients and HVs (p = 0.053).

WSS in the pAAo was lower for native MFS patients vs HVs (0.63 ± 0.12 vs 0.76 ± 0.13 Pa, p < 0.001) and vs the ARS MFS patients (0.63 ± 0.12 vs 0.81 ± 0.16 Pa, p < 0.001), but was not different between ARS patients and HVs (p = 0.215). In the dAAo, WSS was significantly higher for ARS MFS patients vs native MFS patients (0.66 ± 0.15 vs 0.58 ± 0.13 Pa, p = 0.031) and vs HVs (0.66 ± 0.15 vs 0.55 ± 0.14 Pa, p = 0.004).

In the pDAo, WSS was significantly different between all categories, highest for HVs and the lowest for ARS MFS patients (healthy: 1.07 ± 0.18, native MFS: 0.88 ± 0.23, ARS MFS: 0.76 ± 0.20 Pa, p < 0.001). In the dDAo, WSS was lower for ARS MFS patients vs HVs (0.91 ± 0.20 vs 1.08 ± 0.21 Pa, p = 0.002).

PWV was higher for both native MFS patients vs HVs (9.0 ± 3.5 vs 6.6 ± 1.4 m/s, p < 0.001) and for ARS MFS patients vs HVs (10.0 ± 2.7 vs 6.6 ± 1.4 m/s, p < 0.001), but was not significantly different between MFS categories (p = 0.220).

All local aortic diameter adjusted analyses showed an overall similar trend compared to the uncorrected values, except for the following parameters:

Diameter adjusted displacement was significantly different between all three categories in the pAAo (healthy: 32.4 ± 5.1 vs native MFS: 29.5 ± 7.0 vs ARS MFS: 18.2 ± 5.2 mm * 100/mm, p < 0.001) and dDAo (healthy: 7.6 ± 1.9 vs native MFS: 6.7 ± 2.1 vs ARS MFS: 4.8 ± 1.8 mm * 100/mm, p < 0.001). Furthermore, diameter-adjusted velocity in the pDAo was significantly lower for native MFS vs ARS MFS patients (2.40 ± 0.68 vs 2.79 ± 0.70 cm/s/mm, p = 0.032) and was significantly higher for HVs vs native MFS patients in the dDAo (3.59 ± 0.76 vs 3.04 ± 0.69 cm/s/mm, p < 0.001). Diameter adjusted dAAo WSS was not significantly different between groups, while diameter adjusted pDAo and dDAo WSS were significantly different between all groups: pDAo, healthy: 46.69 ± 11.93 vs native MFS: 35.55 ± 12.57 vs ARS MFS: 27.74 ± 10.39 mPa/mm), p < 0.001; dDAo, healthy: 47.30 ± 12.95 vs native MFS: 40.13 ± 10.96 vs ARS MFS: 32.80 ± 9.04 mPa/mm, p < 0.001. All diameter-adjusted values are presented in Supplementary Table S1.

### 3.3 Heatmaps and incidence maps

There was no significant difference between pre-post registration displacement values across ROIs used for creating the displacement 3D averages, with a mean difference of −0.057 mm (95%CI: −0.120, 0.005, p = 0.071). However, the post-registration WSS was lower compared to pre-registration with a mean difference of −0.005 Pa (95%CI: −0.009, −0.001), p = 0.012.

The registration of the control geometry template to the individual patient MFS registries resulted in a difference of 0.102 mm (95%CI: 0.050, 0.155) displacement and −0.005 Pa (95%CI: −0.007, −0.003) in WSS after registration.

An overview of the quantification of abnormal displacement and WSS is presented in Table 3. Areas with decreased displacement (% of total surface) were larger for ARS MFS patients in pAAo (p < 0.001), dAAo (p = 0.005), and dDAo (p < 0.001), while areas with increased displacement were not significantly different between MFS categories. Area (% of total surface) of abnormally increased WSS was higher in the pAAo (p < 0.001) and dAAo (p < 0.001) of ARS MFS patients, and areas (% of total surface) with abnormally decreased WSS were also larger for ARS MFS patients in the dAAo (p = 0.002), pDAo (p = 0.006), and dDAo (p = 0.012). Total area of abnormally directed WSS (% of total surface) was higher for ARS MFS patients in the pAAo (p = 0.006) and dAAo (p = 0.004).

**Table 3:** Quantification and comparison of abnormal wall shear stress and displacement.

	Increased			Decreased		
	Native MFS (n = 50)	ARS MFS (n = 32)	p-value	Native MFS (n = 50)	ARS MFS (n = 32)	p-value
*Displacement*						
Proximal ascending aorta, (% of total area)	0 (IQR: 0–1)	0 (IQR: 0–0)	0.033	1 (IQR: 0–36)	83 (IQR: 68–91)	**<0.001**
Distal ascending aorta, (% of total area)	0 (IQR: 0–22)	0 (IQR: 0–6)	0.491	0 (IQR: 0–1)	4 (IQR: 0–24)	**0.005**
Proximal descending aorta, (% of total area)	0 (IQR: 0–9)	4 (IQR: 0–24)	0.062	0 (IQR: 0–1)	0 (IQR: 0–2)	0.316
Distal descending aorta, (% of total area)	0 (IQR: 0–3)	0 (IQR: 0–7)	0.640	0 (IQR: 0–4)	14 (IQR: 1–22)	**<0.001**
*Wall shear stress*						
Proximal ascending aorta, (% of total area)	1 (IQR: 0–2)	12 (IQR: 2–18)	**<0.001**	4 (IQR: 2–8)	5 (IQR: 1–8)	0.853
Distal ascending aorta, (% of total area)	2 (IQR: 1–5)	11 (IQR: 7–21)	**<0.001**	0 (IQR: 0–1)	1 (IQR: 0–2)	**0.002**
Proximal descending aorta, (% of total area)	0 (IQR: 0–1)	0 (IQR: 0–2)	0.425	5 (IQR: 2–9)	10 (IQR: 4–19)	**0.006**
Distal descending aorta, (% of total area)	1 (IQR: 1–3)	1 (IQR: 0–3)	0.631	1 (IQR: 0–3)	2 (IQR: 1–5)	**0.012**
*Abnormally directed WSS*	*Abnormal vector direction*					
Proximal ascending aorta, (% of total area)	6 (IQR: 3–10)	9 (IQR: 6–16)	**0.006**			
Distal ascending aorta, (% of total area)	8 (IQR: 6–10)	12 (IQR: 7–16)	**0.004**			
Proximal descending aorta, (% of total area)	4 (IQR: 3–7)	5 (IQR: 4–9)	0.129			
Distal descending aorta, (% of total area)	6 (IQR: 4–10)	9 (IQR: 5–11)	0.067			

*Native MFS* Marfan syndrome patient without a history of aortic root surgery, *ARS MFS* Marfan syndrome patient with a history of aortic root surgery, *IQR* interquartile range

Values expressed as median (IQR), p-values of the Wilcoxon signed rank test. A p-value <0.0125 was considered statistically significant

**Fig. 3:**
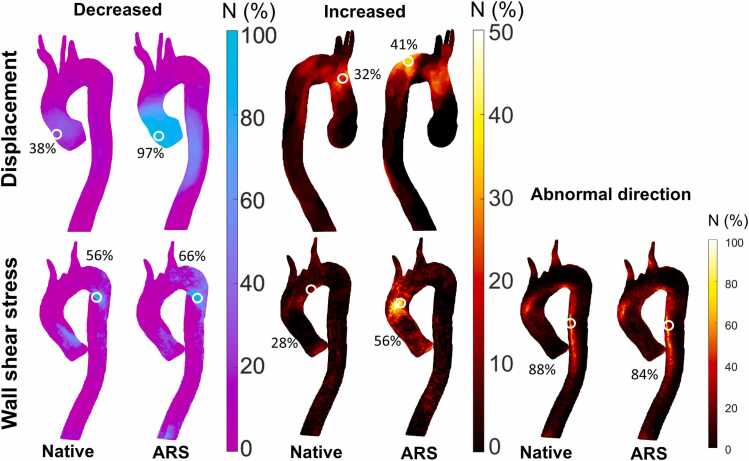
Incidence maps for abnormal displacement and WSS Incidence maps for increased and decreased displacement and wall shear stress (WSS) and abnormally directed WSS for patients without (native) and with a history of aortic root surgery (ARS MFS). The location with the maximum percentage of patients with abnormal displacement and WSS is indicated. *MFS* Marfan syndrome patient, *ARS* aortic root surgery

Fig. 3 shows incidence maps of abnormal displacement and WSS. Decreased displacement is seen throughout the pAAo in ARS MFS patients and, to a lesser extent, in native MFS patients. Increased displacement occurs mainly in the outer curvature of the pDAo in ARS MFS patients and is also frequent in the dAAo of native MFS patients. Both groups show decreased WSS in the inner curvature of the pDAo and abnormally directed WSS throughout the DAo. Increased WSS is mainly found in the dAAo of ARS MFS patients.

An overview of the incidence of at least one voxel with abnormal CMR parameters per ROI and MFS category is presented in Supplementary Table S2. The incidence of abnormal displacement ranged from 13% (4/32) with at least one surface point of increased displacement in the pAAo of ARS MFS patients, to 97% (31/32) with decreased displacement in the pAAo in the same patient group. Incidence of at least one voxel with decreased or increased or abnormally directed WSS was high, ranging between 72% (23/32) for decreased WSS in the pDAo of ARS MFS patients to 100% for several other ROIs and groups.

### 3.4 Correlations and regression

An overview of the correlations between regional displacement, velocity, WSS, and global PWV is presented in Table 4.

**Table 4:** Pearson correlation of regional displacement with regional 4D flow CMR parameters per study group.

	Healthy volunteers (n = 45)		Native MFS (n = 50)		ARS MFS (n = 32)	
	r [95% CI]	p-value	r [95% CI]	p-value	r [95% CI]	p-value
*Velocity*						
Proximal ascending aorta	0.33 [0.04, 0.57]	**0.026**	0.52 [0.28, 0.70]	**<0.001**	−0.04 [−0.38, 0.32]	0.841
Distal ascending aorta	0.12 [−0.18, 0.40]	0.418	0.21 [−0.07, 0.46]	0.143	0.10 [−0.26, 0.43]	0.587
Proximal descending aorta	0.26 [−0.04, 0.51]	0.085	0.34 [0.07, 0.57]	**0.015**	0.42 [0.08, 0.67]	**0.017**
Distal descending aorta	0.35 [0.06, 0.58]	**0.019**	0.40 [0.13, 0.61]	**0.004**	0.23 [−0.13, 0.54]	0.200
*WSS*						
Proximal ascending aorta	0.37 [0.09, 0.60]	**0.012**	0.39 [0.13, 0.60]	**0.005**	−0.12 [−0.45, 0.24]	0.511
Distal ascending aorta	0.11 [−0.19, 0.39]	0.486	0.20 [−0.08, 0.46]	0.154	0.18 [−0.18, 0.50]	0.332
Proximal descending aorta	0.15 [−0.15, 0.43]	0.316	0.21 [−0.07, 0.46]	0.142	0.46 [0.13, 0.70]	**0.008**
Distal descending aorta	0.28 [−0.02, 0.53]	0.063	0.31 [0.04, 0.55]	**0.026**	0.27 [−0.09, 0.56]	0.138
*Global PWV*						
Proximal ascending aorta*	−0.17 [−0.44, 0.13]	0.254	−0.30 [−0.53, −0.02]	**0.035**	−0.18 [−0.50, 0.18]	0.326
Distal ascending aorta*	−0.34 [−0.57, −0.05]	**0.024**	−0.29 [−0.53, −0.02]	**0.039**	−0.16 [−0.48, 0.20]	0.378
Proximal descending aorta*	−0.49 [−0.68, −0.23]	**<0.001**	−0.32 [−0.55, −0.05]	**0.023**	−0.26 [−0.56, 0.10]	0.150
Distal descending aorta*	−0.21 [−0.47, 0.09]	0.176	−0.09 [−0.36, 0.19]	0.514	0.25 [−0.11, 0.55]	0.176

*Native MFS* Marfan syndrome patient without a history of aortic root surgery, *ARS MFS* Marfan syndrome patient with a history of aortic root surgery, *PWV* pulse wave velocity, *WSS* wall shear stress, *4D* four-dimensional, *CMR* cardiovascular magnetic resonance, *CI* confidence interval

A p-value <0.05 was considered statistically significant

Data are Pearson correlation coefficients [95% confidence interval]

* Correlation between global pulse wave velocity and regional displacement

Mean displacement showed a moderate positive correlation with velocity in the pAAo of native MFS patients (r = 0.52, p < 0.001) and in HVs (r = 0.33, p = 0.026). No such association was observed in ARS MFS (p = 0.841). Additionally, significant positive relationships between velocity and displacement were found in the pDAo of native MFS (r = 0.34, p = 0.015) and ARS MFS (r = 0.42, p = 0.017), as well as in the dDAo of HVs (r = 0.35, p = 0.019) and native MFS (r = 0.40, p = 004). For WSS, displacement correlated positively in the pAAo of HVs (r = 0.37, p = 0.012) and native MFS patients (r = 0.39, p = 0.005). In the pDAo, WSS was significantly related to displacement only in ARS MFS (r = 0.46, p = 0.008). Lastly, a significant correlation was observed between WSS and displacement in the dDAo in native MFS (r = 0.31, p = 0.026). Global PWV was inversely associated with displacement in multiple regions in HVs and native MFS patients. The strongest relationship was seen in the pDAo of HVs (r = −0.49).

Univariable logistic regression showed that a suspected haploinsufficient *FBN1* gene variant was associated with higher odds of abnormally increased pDAo displacement (odds ratio: 3.06 (95% CI: 1.11, 9.42), p = 0.038). Associations with ARS, sex, medication use, age, body surface area, and blood pressure were not statistically significant (Supplementary Table S3).

## 4 Discussion

In the current study, we leveraged CMR capabilities by acquiring 4D bSSFP and 4D flow CMR data within the same examination, enabling a comprehensive assessment of aortic hemodynamics and biomechanics and their interaction.

We found a statistically significant correlation between displacement and velocities at several aortic levels across all three study groups. This suggests that higher kinetic energy (defined as one-half the mass times velocity squared, though not separately calculated in this study) is likely to result in a greater force exerted on the aortic wall, leading to higher displacement values. This was previously observed in a fluid-structure interaction simulation study modeling stenotic arteries with flexible aortic walls, where the highest displacement was also seen at higher velocities [34].

ARS has previously been identified as (independent) risk factor for TBAD in MFS [3], [5], [6], [35]. If these findings reflect a direct effect of ARS rather than ARS merely serving as a marker of vascular disease severity, alterations in flow and displacement characteristics are expected following surgery that may affect aortic tissue properties.

Our data showed that MFS patients, both with and without prior ARS, exhibited flow and displacement abnormalities. While some of these abnormalities existed in both MFS groups (abnormally decreased WSS in the inner DAo and abnormally directed WSS throughout the DAo), incidence maps of ARS MFS patients also demonstrated distinct features: lower displacement in the AAo, higher WSS in the dAAo, and localized increased displacement on the outside of the pDAo. While the latter was also visible to some extent in the native MFS patients, there was a trend toward a higher area for ARS MFS patients. Flow and displacement differences in the AAo likely result from the implanted graft, which has different biomechanical properties than native aortic tissue [36]. Our data suggest that the surgery also affects the DAo, as the stiff graft in the AAo can no longer buffer hemodynamic changes effectively [37]. In a replaced root, this loss of elasticity may increase pressure fluctuations in the DAo, contributing to greater motion and dilatation, reflected by the regionally higher pDAo displacement and diameter observed in ARS MFS patients [6]. Similarly, a study in porcine aortas showed increased wall pressure and pressure change rates after prosthetic AAo placement [38].

The fact that a similar pattern of decreased pAAo displacement accompanied by increased DAo displacement was observed in native MFS patients may indicate an increase in AAo vascular stiffening in the native MFS patients, though less pronounced compared to ARS MFS patients. This is supported by the negative association with PWV, an indirect measure of arterial stiffness, observed in both native and ARS MFS patients, suggesting that aortic displacement may serve as a novel marker of arterial stiffness [39].

We observed increased velocity in the AAo and decreased velocity in the DAo of ARS MFS patients, even after correction for the local aortic diameter. The switch from increased to decreased velocity and WSS from AAo to DAo of ARS MFS patients, previously reported by others, indicates a loss of kinetic energy in blood flow through the aorta [11], [40], [41], [42]. This energy loss likely results from inefficient flow patterns after surgery, as the graft and altered aortic morphology expose the aorta to a different pressure distribution [6], [40]. Indeed, in non-MFS patients with prior root surgery, flow deviation in the AAo occurred four times more often than in HVs, mainly near the distal anastomosis kink [40]. In MFS, such abnormal flow patterns have been reported in both the AAo and inner DAo [24], [42], [43].

If these flow abnormalities occur near the aortic wall, they will result in the abnormally directed WSS. Indeed, we found abnormally directed WSS in nearly all MFS patients (except in the pDAo of one ARS MFS patient), with a larger affected area in the AAo of ARS MFS patients compared to native MFS. In the DAo, abnormally directed WSS was also observed, but without differences between groups, suggesting it is not a direct result of root surgery. In contrast, WSS magnitude was lower in the pDAo of ARS MFS patients and most often located in the inner curvature, corresponding to a known predilection site for TBAD [8], [10], [42]. Abnormal WSS may promote aortic wall remodeling through endothelial mechano-transduction [40].

Incidence maps showed locally increased displacement in the outer pDAo in nearly half of ARS MFS patients and was associated with presence of a haploinsufficient *FBN1* variant, a known risk factor for poorer prognosis in MFS [44]. This localized deformation may reflect increased dissipation of kinetic energy within the aortic wall. It has been found that energy loss is correlated with local media degeneration and imbalances in elastin and collagen composition [45]. Examining tissue composition in the outer pDAo of patients with local displacement abnormalities could therefore provide valuable context, in case any of these patients would require DAo surgery. However, conclusively establishing a link between abnormal displacement, WSS alterations, and TBAD development would require longitudinal studies with an adequate number of aortic events for analysis, a task that is particularly challenging in rare diseases, where clinical events are infrequent.

## 5 Limitations

We observed small but significant differences in parameter values after registration, likely due to variations in the position of ROIs based on anatomical landmarks, which will differ slightly in position between patients. As these post-registration differences were generally minor, their impact on the results is expected to be limited.

## 6 Conclusion

Aortic displacement and flow characteristics are abnormal in MFS patients both with and without prior ARS. However, the patterns and magnitude of these abnormalities differ between groups, suggesting they are influenced by the surgical intervention. Whether these changes contribute to subsequent aortic dilatation or dissection remains to be determined in longitudinal studies.

## Funding

This study is part of the research program Applied and Engineering Sciences and the project Comprehensive Assessment of 4D Thoracic Aorta Biomechanics Using Novel Cardiac MRI Technology (number 18402), financed by the Dutch Research Council (NWO). E.M.S. acknowledges funding by 10.13039/100011950ITEA Eureka cluster on Software innovation through the SIGNET project number 20052.

## Declaration of Generative AI and AI-assisted technologies in the writing process

During the preparation of this work, the authors used Grammarly and ChatGPT to correct English grammar/spelling and improve readability. After using this tool/service, the authors reviewed and edited the content as needed and take full responsibility for the content of the publication.

## Declaration of competing interests

The authors declare that they have no known competing financial interests or personal relationships that could have appeared to influence the work reported in this paper.

## Data Availability

For this study, we used the Amsterdam UMC “PROspective Undersampling in multiple Dimensions” (PROUD) software patch (https://mriresearch.amsterdam/software/aumcproudpatch/). All data and software are available on reasonable request.

## Footnotes

Appendix A Supplementary data associated with this article can be found in the online version at doi:10.1016/j.jocmr.2026.102731.
