# Supplementary material for: Aortic displacement and hemodynamics are abnormal in patients with Marfan syndrome: A combined four-dimensional balanced steady-state free precession and four-dimensional flow cardiac magnetic resonance study
Source: J Cardiovasc Magn Reson. 2026 Apr 20;28(1):102731. doi: 10.1016/j.jocmr.2026.102731 (PMC13237544; doi:10.1016/j.jocmr.2026.102731)
Supplement: Supplementary file 2 — Supplementary material [file mmc1.docx]

# Supplementary material

|  | Healthy (n=45) | Native MFS (n=50) | ARS MFS  (n=32) |  | Native MFS-Healthy | ARS MFS-Healthy | ARS MFS-Native MFS |
| --- | --- | --- | --- | --- | --- | --- | --- |
|  | **Mean ± SD** | **Mean ± SD** | **Mean ± SD** | **ANOVA p-value** | ***Tukey p-value*** | ***Tukey p-value*** | ***Tukey p-value*** |
| Displacement |  |  |  |  |  |  |  |
| Proximal ascending aorta (mm*100/mm) | 32.4 ± 5.1 | 29.5 ± 7.0 | 18.2 ± 5.2 | **<0.001** | **0.049** | **<0.001** | **<0.001** |
| Distal ascending aorta (mm*100/mm) | 14.9 ± 3.7 | 15.2 ± 4.5 | 13.5 ± 4.0 | 0.175 |  |  |  |
| Proximal descending aorta (mm*100/mm) | 6.4 ± 1.9 | 5.7 ± 1.7 | 5.8 ± 2.2 | 0.169 |  |  |  |
| Distal descending aorta (mm*100/mm) | 7.6 ± 1.9 | 6.7 ± 2.1 | 4.8 ± 1.8 | **<0.001** | **0.047** | **<0.001** | **<0.001** |
| Velocity |  |  |  |  |  |  |  |
| Proximal ascending aorta (cm/s) | 2.43 ± 0.47 | 2.13 ± 0.41 | 2.45 ± 0.42 | **0.001** | **0.003** | 0.974 | **0.004** |
| Distal ascending aorta (cm*s^-1^mm^-1^) | 2.08 ± 0.57 | 2.07 ± 0.52 | 2.18 ± 0.43 | 0.625 |  |  |  |
| Proximal descending aorta (cm*s^-1^mm^-1^) | 3.34 ± 0.62 | 2.79 ± 0.72 | 2.40 ± 0.68 | **<0.001** | **<0.001** | **<0.001** | **0.032** |
| Distal descending aorta (cm*s^-1^mm^-1^) | 3.59 ± 0.76 | 3.04 ± 0.69 | 2.67 ± 0.67 | **<0.001** | **<0.001** | **<0.001** | 0.057 |
| WSS |  |  |  |  |  |  |  |
| Proximal ascending aorta (mPa/mm) | 25.32 ± 6.05 | 20.73 ± 5.41 | 25.45 ± 5.84 | **<0.001** | **<0.001** | **0.994** | **0.001** |
| Distal ascending aorta (mPa/mm) | 18.65 ± 6.34 | 19.21 ± 5.99 | 20.76 ± 5.46 | 0.304 |  |  |  |
| Proximal descending aorta (mPa/mm) | 46.69 ± 11.93 | 35.55 ± 12.57 | 27.74 ± 10.39 | **<0.001** | **<0.001** | **<0.001** | **0.012** |
| Distal descending aorta (mPa/mm) | 47.30 ± 12.95 | 40.13 ± 10.96 | 32.80 ± 9.04 | **<0.001** | **0.007** | **<0.001** | **0.013** |

**Table S1.** 4D bSSFP and 4D flow CMR values adjusted for local aortic diameter.

*Parameters expressed as mean +/- SD. Native MFS: Marfan syndrome patient without a history of aortic root surgery, ARS MFS: Marfan syndrome patient with a history of aortic root surgery*

**Table S2.** Incidence of at least one voxel of abnormal displacement and WSS voxel per region per MFS group

|  | Elevated | | | Decreased | | |
| --- | --- | --- | --- | --- | --- | --- |
|  | **Native MFS (n=50)** | **ARS** **MFS**  **(n=32)** | **p-value** | **Native MFS (n=50)** | **ARS** **MFS**  **(n=32)** | **p-value** |
| Displacement |  |  |  |  |  |  |
| Proximal ascending aorta,  n (%) | 17 (34%) | 4 (13%) | 0.030 | 27 (54%) | 31 (97%) | **<0.001** |
| Distal ascending aorta,  n (%) | 27 (54%) | 16 (50%) | 0.723 | 16 (32%) | 19 (59%) | **0.014** |
| Proximal descending aorta,  n (%) | 27 (54%) | 21 (66%) | 0.297 | 21 (42%) | 17 (53%) | 0.324 |
| Distal descending aorta,  n (%) | 23 (46%) | 16 (50%) | 0.723 | 23 (46%) | 28 (88%) | **<0.001** |
| WSS |  |  |  |  |  |  |
| Proximal ascending aorta,  n (%) | 50 (100%) | 32 (100%) | 1.000 | 50 (100%) | 32 (100%) | 1.000 |
| Distal ascending aorta,  n (%) | 47 (94%) | 32 (100%) | 0.158 | 44 (88%) | 32 (100%) | 0.042 |
| Proximal descending aorta,  n (%) | 42 (84%) | 23 (72%) | 0.186 | 48 (96%) | 32 (100%) | 0.252 |
| Distal descending aorta,  n (%) | 48 (96%) | 31 (97%) | 0.837 | 49 (98%) | 32 (100%) | 0.421 |
| Abnormally directed WSS | **Abnormal vector direction** | | |  |  |  |
| Proximal ascending aorta,  n (%) | 50 (100%) | 32 (100%) | 1.000 |  |  |  |
| Distal ascending aorta,  n (%) | 50 (100%) | 32 (100%) | 1.000 |  |  |  |
| Proximal descending aorta,  n (%) | 49 (98%) | 32 (100%) | 0.421 |  |  |  |
| Distal descending aorta,  n (%) | 50 (100%) | 32 (100%) | 1.000 |  |  |  |

*Native MFS: Marfan syndrome patient without a history of aortic root surgery, ARS MFS: Marfan syndrome patient with a history of aortic root surgery*

**Table S3.** Logistic regression for incidence of elevated displacement in the proximal descending aorta

| Univariate logistic regression | | |
| --- | --- | --- |
| Predictor | **OR (95% CI)** | **p-value** |
| Male sex | 1.24 (0.51, 3.03) | 0.628 |
| Age (years) | 0.98 (0.93, 1.03) | 0.378 |
| BSA (m^2^) | 0.87 (0.12, 6.41) | 0.886 |
| Haploinsufficient FBN1 mutation | 3.06 (1.11, 9.42) | **0.038** |
| History of aortic root surgery | 1.63 (0.66, 4.16) | 0.299 |
| Betablocker use | 2.47 (1.00, 6.34) | 0.053 |
| ARB use | 2.31 (0.95, 5.77) | 0.068 |
| Betablocker + ARB | 2.90 (1.00, 9.77) | 0.063 |
| Systolic BP (mmHg) | 1.01 (0.97, 1.05) | 0.669 |
| Diastolic BP (mmHg) | 1.00 (0.94, 1.06) | 0.905 |
| Pulse pressure (mmHg) | 1.03 (0.96, 1.10) | 0.421 |

*ARB Angiotensin II receptor blocker, BP: blood pressure, BSA: body surface area, OR: odds ratio, CI: confidence interval.*
